# Neurodevelopmental disorders as a risk factor for temporomandibular disorder: evidence from Mendelian randomization studies

**DOI:** 10.3389/fgene.2024.1365596

**Published:** 2024-03-08

**Authors:** Xueqiang Wu, Zefang Li, Yiping Cui, Zhaojun Yan, Tingting Lu, Song Cui

**Affiliations:** ^1^ Department of Health Science, Shandong University of Traditional Chinese Medicine, Jinan, China; ^2^ Department of the First Clinical Medicine, Shandong University of Traditional Chinese Medicine, Jinan, China; ^3^ Affiliated Hospital of Shandong University of Traditional Chinese Medicine, Jinan, China

**Keywords:** neurodevelopmental disorders, temporomandibular disorder, Mendelian randomization, attention deficit and hyperactivity disorder, tourette disorder, austim spectrum disorder

## Abstract

**Objective:** This study aims to clarify the incidence rate of temporomandibular joint disease in patients with mental disorders.

**Methods:** Data extracted from the Psychiatric Genomics Consortium and FinnGen databases employed the Mendelian Randomization (MR) method to assess the associations of three neurodevelopmental disorders (NDDs)—Attention-Deficit/Hyperactivity Disorder (ADHD), Autism Spectrum Disorder (ASD), and Tourette’s Disorder (TD)—as exposure factors with Temporomandibular Disorder (TMD). The analysis used a two-sample MR design, employing the Inverse Variance Weighted (IVW) method to evaluate the relationships between these disorders and Temporomandibular Disorder. Sensitivity analysis and heterogeneity assessments were conducted. Potential confounding factors like low birth weight, childhood obesity, and body mass index were controlled for.

**Results:** The study found that ADHD significantly increased the risks for TMD (OR = 1.2342, 95%CI (1.1448–1.3307), *p* < 0.00001), TMD (including avohilmo) (OR = 1.1244, 95%CI (1.0643–1.1880), *p* = 0.00003), TMD-related pain (OR = 1.1590, 95%CI (1.0964–1.2252), *p* < 0.00001), and TMD-related muscular pain associated with fibromyalgia (OR = 1.1815, 95%CI (1.1133–1.2538), *p* < 0.00001), while other disorders did not show significant causal relationships.

**Conclusion:** This study reveals the elevated risk of various TMD aspects due to ADHD. Furthermore, we discuss the link between low vitamin D levels ADHD and TMD. Future research should address these limitations and delve further into the complex interactions between ADHD, ASD, TD, and TMD.

## 1 Introduction

Temporomandibular Disorder (TMD) is a complex clinical condition characterized by a diverse range of symptoms, including restricted lower jaw movement, muscle pain, discomfort in the temporomandibular joint, joint sounds, systemic myofascial pain, and irregularities and limitations in jaw movement (Durham et al., 2015). In the healthcare sector, the application of TMD has seen disproportionate growth, making it a primary factor in non-dental facial and oral pain (Slade et al., 2013; Lövgren et al., 2015; Slade et al., 2016).

The etiology of TMD involves various factors, encompassing biology, environment, social, emotional, and cognitive elements, all interacting with each other (Chisnoiu et al., 2015; Svensson and Kumar, 2016; Fillingim et al., 2018; Shrivastava et al., 2021). For nearly all TMD patients, conservative treatments are the preferred approach. This includes behavior correction, physical therapy, pharmacological intervention, and the use of jaw appliances aimed at alleviating muscle tension, adjusting jaw position, and reducing joint burden (Durham et al., 2015; Wieckiewicz et al., 2015).

TMD can not only lead to a decrease in work efficiency but may also trigger issues related to the psychological wellbeing of patients, imposing a burden on individuals and causing a certain impact on the overall socio-economic landscape (Cao et al., 2020). Additionally, TMD patients often require multidisciplinary medical services, involving oral health professionals, neurologists, and others, presenting challenges in the rational utilization of healthcare resources.

Neurodevelopmental disorders (NDDs) have an exceptionally high prevalence in the population, and the incidence of this category of disorders is on the rise. As of 2016, there is approximately one case of NDDs for every six children in the United States (Zablotsky et al., 2019). Among them, Attention-Deficit/Hyperactivity Disorder (ADHD) stands out as one of the most common neurodevelopmental disorders during childhood, characterized by symptoms such as difficulty concentrating and impulsive behavior (American Psychiatric Association, 2013; Faraone et al., 2015; Agnew-Blais et al., 2016; Thapar and Cooper, 2016). On the other hand, Autism Spectrum Disorder (ASD) manifests with challenges in social communication, restricted interests, and repetitive behaviors (American Psychiatric Association, 2013). In contrast, Tourette’s Disorder (TD) is identified by persistent vocal and motor tics lasting for over a year (Groth et al., 2017; Robertson et al., 2017). Treatment approaches encompass various methods, including pharmacological intervention and physical support (Díaz-Caneja et al., 2021; Ribas et al., 2023).

The etiology of NDDs involves a complex interplay of genetic, environmental, prenatal, and perinatal factors (Han et al., 2021; Doi et al., 2022). Furthermore, NDDs are associated with numerous other diseases. It is noteworthy that individuals with NDDs have relatively limited access to medical resources, underscoring the need for measures to promote the health, development, and future wellbeing of this patient population.

Research suggests a higher prevalence of TMD in individuals with ADHD, and there is a positive correlation between symptoms of ADHD in adults and TMD (Stelcer et al., 2022). Recent studies also indicate a significant genetic risk overlap among ADHD, ASD, and TD (Agnew-Blais et al., 2016), raising reasonable suspicions of their association with TMD, including TMD-related pain and TMD muscular pain linked with fibromyalgia. However, existing research is susceptible to bias in understanding the relationships between ADHD, ASD, TD, and various aspects of TMD. Currently, no formal studies have assessed the causal relationships between these conditions.

This study aims to use Mendelian randomization methods, conducting a bidirectional analysis using data from the Psychiatric Genomics Consortium (PGC) and the FinnGen database. The objective is to gain a deeper understanding of the complex interactions between NDDs and TMD-related diseases, providing a new perspective for intervening in these conditions.

## 2 Methods

### 2.1 Data sources

In this two-sample Mendelian randomization study, we utilized publicly available databases to investigate three NDDs as exposure factors and their associations with TMD. Ethical approvals were obtained for the original studies conducted.

Genetic association data for ADHD (Demontis et al., 2023), ASD (Grove et al., 2019), and TD (Yu et al., 2019) were sourced from the Psychiatric Genomics Consortium (PGC) database. The study encompassed 38,691 ADHD patients and 186,843 controls, 18,381 ASD patients and 27,969 controls, and 4,819 TS patients and 9,488 controls, all of European descent (Table 1). Diagnosis criteria followed ICD-10 for ADHD and ASD, and DSM-5 criteria for TS. We employed a threshold of P < 5e-06 to screen SNPs associated with NDDs. Secondly, to mitigate the impact of weak instrumental variables on statistical results, we calculated the F-value for each SNP (F = $\frac{\;{\text{beta}}^{2}}{{\text{se}}^{2}}$ ) (Yao et al., 2023) and excluded weak instrumental variables with an F-value below 10 to avoid statistical bias. Additionally, we removed symmetrical SNPs with linkage disequilibrium effects (LD r^2^ < 0.001, clumping distance > 10,000 kb), and purging of palindromic SNPs with allele frequencies approaching 0.5. In the end, we included 194 SNPs as instrumental variables (IVs) for our study (The detail of IVs in Supplementary Table).

**TABLE 1:** Detailed information of the datasets used for Mendelian randomization analyses.

Traits	Year	Authors	Population	Consortium	Sample size	Number of SNPs
ADHD	2023	Havdahl et al	European	PGC	225,534	6774224
ASD	2019	Grove et al	European	PGC	43,350	9,112,386
TS	2019	Yu et al.	European	PGC	14,307	8,265,318
Temporomandibular joint disorders	2023	Kurki et al.	European	Finngen	5,668	20162505
Temporomandibular joint disorders, including avohilmo	2023	Kurki et al.	European	Finngen	13,282	20170236
TMD related pain	2023	Kurki et al.	European	Finngen	10,303	20170236
TMD muscular pain linked with fibromyalgia	2023	Kurki et al.	European	Finngen	8,995	20170236

Data on TMD, including various aspects, were extracted from the FinnGen dataset (Kurki et al., 2023), specifically the R9 version released on 11 May 2023. Diagnosis criteria followed ICD-10 for TMD, TMD muscular pain linked with fibromyalgia, and TMD related pain. We applied the same selection criteria as for NDDs instrumental variables, included 48 SNPs as IVs for our study (The detail of IVs in Supplementary Table). Perusterveyden-huollon avohoidon hoitoilmoitus (Avohilmo) is a component of the Social and Healthcare Treatment Notification System (Hilmo). Avohilmo data is instrumental for decision-making, planning, and research purposes (Kurki et al., 2023). Avohilmo reports furnish the latest information on public utilization of services, care status, health issues, and the spread of epidemics in the population, as well as health promotion services, division of responsibilities, and management practices. To bolster the persuasiveness of our research results, we have incorporated data from two TMD cohorts.

Notably, low birth weight has been correlated with ADHD (Momany et al., 2018). The rising prevalence of childhood obesity presents a significant public health concern, characterized by a complex relationship with adult mental health (He et al., 2023). Additionally, research has revealed a connection between body mass index (BMI) and TMD (Wang et al., 2023).

In this study, we considered birth height (van der Valk et al., 2015), birth weight (Warrington et al., 2019), and BMI (Copenhaver et al., 2020) as potential confounding factors. To minimize their impact on outcomes, we excluded SNPs overlapping with confounding factors in the IVs related to NDDs and TMD (SNPs with P < 5e-08), to avoid the impact of horizontal pleiotropy. Information on birth height, birth weight, and children’s BMI was sourced from The Early Growth Genetics (EGG) Consortium.

### 2.2 Statistical analysis

**FIGURE 1:**
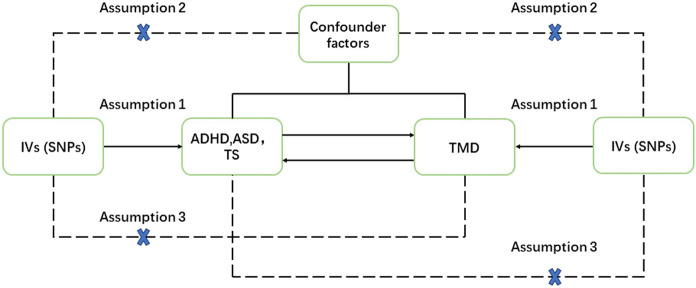
The study framework chart.

The objective of this study was to investigate the bidirectional relationships between ADHD, ASD, TD, and various facets of TMD, encompassing TMD (including avohilmo), TMD-related pain, and TMD muscular pain linked with fibromyalgia. The two-sample Mendelian randomization (MR) design relied on three fundamental assumptions: 1) the strength of association between IVs and the exposures. 2) the absence of any relationship between IVs and unmeasured confounding factors linking exposures and outcomes. 3) IVs exerted their influence solely through the exposures. A rigorous selection process for IVs (with a threshold of P < 5e-06) was employed to minimize any weak correlation between potential confounders and genetic variations (Davey Smith and Hemani, 2014) (Figure 1).

Within this MR study, the primary statistical measure utilized was the inverse variance-weighted (IVW) method, assessing the associations between the three neurodevelopmental disorders and temporomandibular disorders. Additionally, MR-Egger, weighted median, and weighted model approaches were applied.

Sensitivity analysis played a crucial role in detecting potential pleiotropy. Heterogeneity was assessed using the Cochrane Q test, while horizontal pleiotropy was examined via the MR-Egger intercept. Furthermore, leave-one-out analysis was conducted to determine if individual SNPs introduced bias in MR results. In cases of heterogeneity, the primary statistical measure was the random-effects IVW, and in the absence of heterogeneity, the fixed-effects IVW was employed. Multiple testing was addressed through Bonferroni correction, with significance defined as a *p*-value <0.0042 (0.05/12), accounting for three or four exposures and three or four outcomes. *p*-values between 0.0042 and 0.05 were considered suggestive, while *p*-values exceeding 0.05 were regarded as non-significant. All statistical analyses were executed using the TwoSampleMR and MVMR packages in R version 4.2.2. Statistical results were presented as odds ratios and 95% confidence intervals.

## 3 Results

### 3.1 ADHD, ASD, TD, and the causality with TMD, TMD (including avohilmo), TMD related pain, and TMD muscular pain linked with fibromyalgia

The findings of this study, which explored the causal associations between ADHD, ASD, TD, and various aspects of TMD, including TMD (including avohilmo), TMD-related pain, and TMD muscular pain linked with fibromyalgia, are as follows:

After the careful screening for *p*-values and LD clumping, we employed 138, 34, and 22 LD-independent SNPs as IVs for ADHD, ASD, and TD, respectively. After the exclusion of SNPs not present in the outcome variables, the two-sample Mendelian randomization analysis unveiled a significant association between ADHD and TMD (OR = 1.2342, 95%CI (1.1448–1.3307), *p* < 0.00001), TMD (including avohilmo) (OR = 1.1244, 95%CI (1.0643–1.1880), *p* = 0.00003), TMD-related pain (OR = 1.1590, 95%CI (1.0964–1.2252), *p* < 0.00001), and TMD muscular pain linked with fibromyalgia (OR = 1.1815, 95%CI (1.1133–1.2538), *p* < 0.00001). Importantly, even after applying Bonferroni correction (*p* < 0.004), all four results retained their significance. Thus, it was confirmed that ADHD is a risk factor for TMD, TMD (including avohilmo), TMD-related pain, and TMD muscular pain linked with fibromyalgia.

**FIGURE 2:**
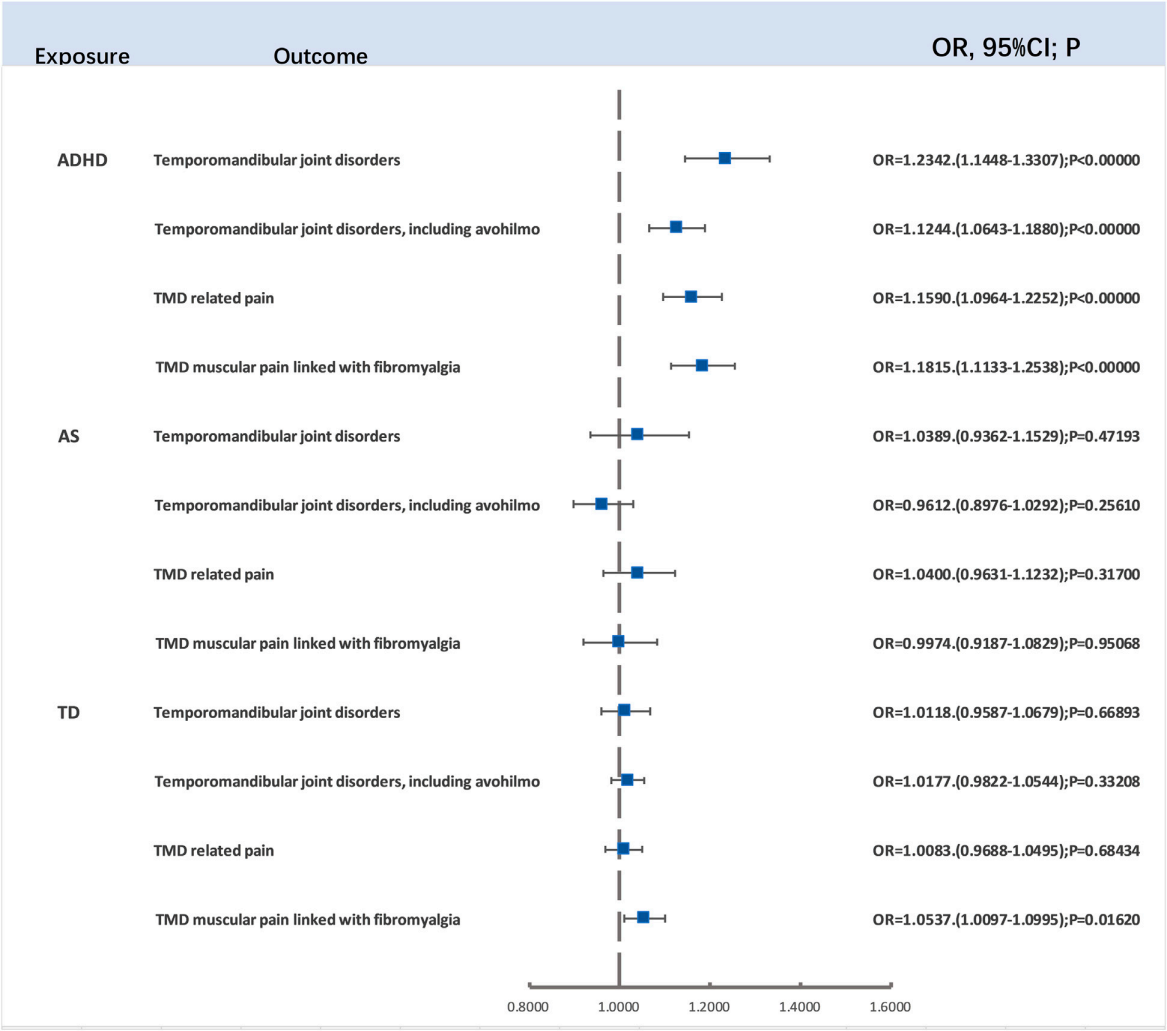
The forest map for Causal Relationship Between ADHD, TS and ASD and TMD.

However, there was no significant relationship between TD and TMD muscular pain linked with fibromyalgia (OR = 1.0537, 95%CI (1.0097–1.0995), *p* = 0.0162) following Bonferroni correction (*p* < 0.004) (Figure 2). Additional details, including results from the Cochran Q test, are available in the Supplementary Materials. The presence of heterogeneity was assessed through MR-Egger and IVW tests, with the corresponding information depicted in a funnel plot. Significantly, no compelling evidence of pleiotropy was detected in the MR-Egger intercept.

### 3.2 The causal relationship between TMD, TMD (including avohilmo), TMD related pain, and TMD muscular pain linked with fibromyalgia and ADHD, ASD, and TD

**FIGURE 3:**
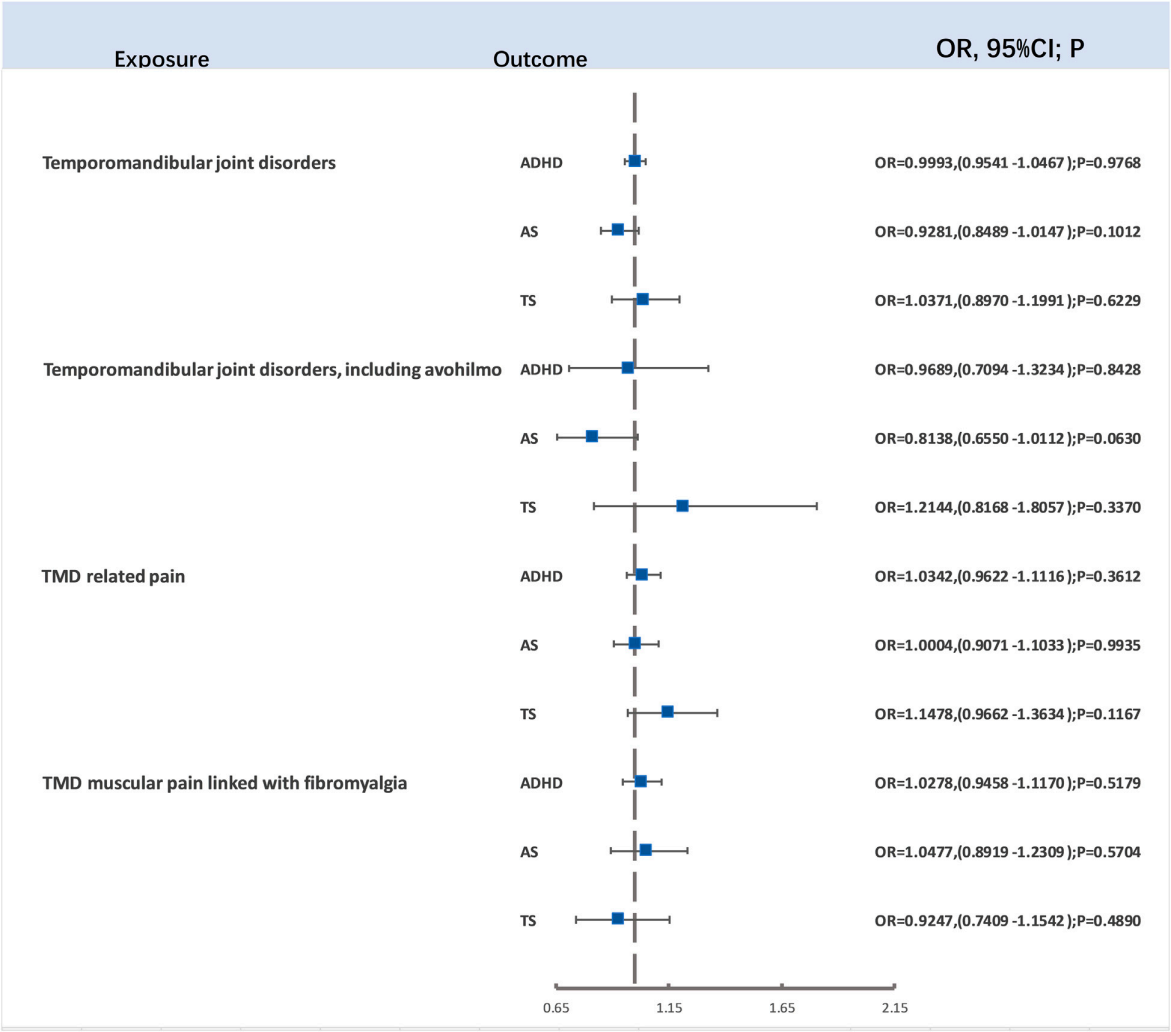
The forest map for Causal Relationship Between TMD and ADHD, TS and ASD.

For evaluating the causal links between TMD, TMD (including avohilmo), TMD related pain, and TMD muscular pain linked with fibromyalgia and ADHD, ASD, and TD, our study employed 16, 5, 15, and 12 SNPs as IVs for TMD, TMD (including avohilmo), TMD related pain, and TMD muscular pain linked with fibromyalgia, respectively. This selection was made following rigorous *p*-value screening and LD clumping. After the removal of SNPs not present in the outcome variables and those displaying F-statistics lower than 10, the two-sample Mendelian randomization analysis did not demonstrate significant associations (Figure 3). Detailed outcomes of heterogeneity and pleiotropy tests can be found in the Supplementary Materials.

## 4 Discussion

To the best of our knowledge, we are the first to comprehensively investigate the intricate relationship between NDDs and TMD based on genetic information. Employing a bidirectional MR approach, we explored the complex associations between three NDDs (ADHD, ASD, and TD) and diseases related to TMD across multiple levels, involving TMD, TMD-related pain, and TMD muscular pain linked with fibromyalgia.

The results of MR analysis revealed a striking discovery: after excluding traditional risk factors and carefully controlling for heterogeneity effects, ADHD significantly increased the risk of TMD, TMD-related pain, and TMD muscular pain linked with fibromyalgia. It is worth noting that reverse MR analysis did not reveal any significant causal relationships. No significant causal relationships were observed between ASD, TD, and TMD.

Currently, the diagnostic criteria for TMD, known as DC/TMD, use a two-axis approach. Axis I encompasses the clinical diagnosis of TMD, while Axis II involves psychological distress and psychosocial functional impairment (Schiffman et al., 2014). This clearly indicates a close connection between psychological and behavioral disorders and TMD (Fillingim et al., 2013; Salinas Fredricson et al., 2022). Studies reveal that anxiety, depression, and ADHD are commonly associated with TMD, sharing certain neurochemical dysregulations and central nervous system changes, such as alterations in the serotonin system and reactivity of the hypothalamic-pituitary-adrenal axis (Jo et al., 2016; Llorens et al., 2022; Jue et al., 2023; Kim et al., 2023). This underscores the intricate relationship between ADHD and TMD.

Ângelo’s research reveals that among TMD patients, 42.88% concurrently suffer from other chronic diseases, with the majority being mental, behavioral, or NDDs (33.76%) (Ângelo et al., 2023). Stelcer’s research indicates that compared to healthy individuals, ADHD patients experience more issues related to muscle tension, TMD, and various physical manifestations (Stelcer et al., 2022). Simultaneously, Mota’s research highlights complex direct and indirect influences among adverse oral habits, sleep bruxism, and ADHD symptoms in school-aged children (Mota-Veloso et al., 2017). Oral functional habits are considered robust predictors for the occurrence of TMD (Ohrbach et al., 2013). In summary, there may be a mutual association between TMD and ADHD. These viewpoints align with the findings of our study.

Winocur’s research dismisses a definite connection between ASD and TMD, asserting that the prevalence of TMD-related pain is low in ASD patients. However, due to the complexity of pain assessment in ASD patients, further research is needed to validate this perspective (Winocur-Arias et al., 2023). This research conclusion aligns with ours, as no causal relationship was found between ASD and TMD.

Ella’s research suggests that Tourette Syndrome (TS) may have profound effects on the oral cavity. They hypothesize that the oro-facial tics and compulsive behaviors observed in individuals with TD, including teeth grinding, tongue movements, jaw activities, and lip biting, may lead to destructive oral damage (Ella et al., 2017). As of now, no studies have been found regarding the correlation between TD and TMD. Our research findings, from a genetic perspective, negate the association between TS and TMD, providing a valuable supplement to existing studies.

Vitamin D Deficiency is associated with various developmental disorders, with a particular study noting that individuals with TMD often have lower serum vitamin D levels (Ferrillo et al., 2022). Based on these studies, we hypothesized that vitamin D might play a mediating role between ADHD and TMD. However, our two-step mediation analysis did not yield positive results. Our research indicates that despite the complex relationships among ADHD, TMD, and vitamin D, vitamin D does not have an effect on increasing the risk of TMD in individuals with ADHD (Detailed results in the Supplementary Table).

To the best of our knowledge, we are the first to delve into the relationship between NDDs and TMD from a genetic perspective. The research findings reveal a significant association between ADHD and diseases related to TMD, providing a novel perspective on our understanding of the connection between NDDs and TMD. This discovery holds potential guidance for clinical practice and the formulation of treatment plans. The detailed analysis offers clues to the potential biological mechanisms among related diseases, paving the way for future in-depth investigations.

The scope of this study is subject to several limitations. Firstly, we did not explore the stratified effects of age, health status, or gender differences. Additionally, excluding SNPs related to known confounding factors may not fully address unknown confounding factors influencing the association between ADHD and TMD. Finally, our study only included research participants of European descent, and considering that disease patterns may exhibit lineage-related variations, the lack of broader applicability to different populations is acknowledged. Future research can target diverse populations, delving into the impact of age, gender, and health status on the relationship between NDDs and TMD. For the ADHD-TMD relationship we discovered, future research could delve into the underlying genetic mechanisms.

Based on the research findings, early screening and intervention measures should be established to alleviate symptoms and improve the quality of life for patients. This includes regular oral examinations and behavioral assessments for school-aged children. Implementing public health education programs to raise awareness of NDDs and TMD is crucial. This helps reduce societal misconceptions about these diseases, promoting early detection and treatment. Simultaneously, advancing interdisciplinary teamwork involving neurologists, dentists, and psychologists can better address the comprehensive needs of patients.

## 5 Conclusion

This study reveals the elevated risk of various TMD aspects due to ADHD. Furthermore, we discuss the link between low vitamin D levels, ADHD and TMD. Future research should address these limitations and delve further into the complex interactions between ADHD, ASD, TD, and TMD.

## Data Availability

The original contributions presented in the study are included in the article/Supplementary Material, further inquiries can be directed to the corresponding author.

## References

[B1] Agnew-Blais J. C. Polanczyk G. V. Danese A. Wertz J. Moffitt T. E. Arseneault L. (2016). Evaluation of the persistence, remission, and emergence of attention-deficit/hyperactivity disorder in young adulthood. JAMA Psychiatry 73, 713–720. 10.1001/jamapsychiatry.2016.0465 27192174 PMC5475268

[B2] American Psychiatric Association (2013). Diagnostic and statistical manual of mental disorders: DSM-5™. 5th ed. Arlington, VA, US: American Psychiatric Publishing, Inc., 947. xliv.

[B3] Ângelo D. F. Mota B. João R. S. Sanz D. Cardoso H. J. (2023). Prevalence of clinical signs and symptoms of temporomandibular joint disorders registered in the eurotmj database: a prospective study in a Portuguese center. J. Clin. Med. 12, 3553. 10.3390/jcm12103553 37240658 PMC10219561

[B4] Cao Y. Yap A. U. Lei J. Zhang M. J. Fu K. Y. (2020). Subtypes of acute and chronic temporomandibular disorders: their relation to psychological and sleep impairments. Oral Dis. 27, 1498–1506. 10.1111/odi.13692 33098197

[B5] Chisnoiu R. Picos A. Lascu L. Chisnoiu P. D. Popa S. Picos A. M. (2015). Factors involved in the etiology of temporomandibular disorders - a literature review. Med. Pharm. Rep. 88, 473–478. 10.15386/cjmed-485 PMC4689239 26732121

[B6] Copenhaver G. P. Vogelezang S. Bradfield J. P. Ahluwalia T. S. Curtin J. A. Lakka T. A. (2020). Novel loci for childhood body mass index and shared heritability with adult cardiometabolic traits. PLOS Genet. 16, e1008718. 10.1371/journal.pgen.1008718 33045005 PMC7581004

[B7] Davey Smith G. Hemani G. (2014). Mendelian randomization: genetic anchors for causal inference in epidemiological studies. Hum. Mol. Genet. 23, R89–R98. 10.1093/hmg/ddu328 25064373 PMC4170722

[B8] Demontis D. Walters G. B. Athanasiadis G. Walters R. Therrien K. Nielsen T. T. (2023). Genome-wide analyses of ADHD identify 27 risk loci, refine the genetic architecture and implicate several cognitive domains. Nat. Genet. 55, 198–208. 10.1038/s41588-022-01285-8 36702997 PMC10914347

[B9] Díaz-Caneja C. M. State M. W. Hagerman R. J. Jacquemont S. Marín O. Bagni C. (2021). A white paper on a neurodevelopmental framework for drug discovery in autism and other neurodevelopmental disorders. Eur. Neuropsychopharmacol. 48, 49–88. 10.1016/j.euroneuro.2021.02.020 33781629

[B10] Doi M. Usui N. Shimada S. (2022). Prenatal environment and neurodevelopmental disorders. Front. Endocrinol. 13. 10.3389/fendo.2022.860110 PMC8964779 35370942

[B11] Durham J. Newton-John T. R. O. Zakrzewska J. M. (2015). Temporomandibular disorders. Bmj 350, h1154. 10.1136/bmj.h1154 25767130

[B12] Ella B. Ghorayeb I. Burbaud P. Guehl D. (2017). Bruxism in movement disorders: a comprehensive review. J. Prosthodont. official J. Am. Coll. Prosthodont. 26, 599–605. 10.1111/jopr.12479 27077925

[B13] Faraone S. V. Asherson P. Banaschewski T. Biederman J. Buitelaar J. K. Ramos-Quiroga J. A. (2015). Attention-deficit/hyperactivity disorder. Nat. Rev. Dis. Prim. 1, 15020. 10.1038/nrdp.2015.20 27189265

[B14] Ferrillo M. Lippi L. Giudice A. Calafiore D. Paolucci T. Renò F. (2022). Temporomandibular disorders and vitamin D deficiency: what is the linkage between these conditions? A systematic review. J. Clin. Med. 11, 6231. 10.3390/jcm11216231 36362456 PMC9655046

[B15] Fillingim R. B. Ohrbach R. Greenspan J. D. Knott C. Diatchenko L. Dubner R. (2013). Psychological factors associated with development of TMD: the OPPERA prospective cohort study. J. pain 14, T75–T90. 10.1016/j.jpain.2013.06.009 24275225 PMC3855656

[B16] Fillingim R. B. Slade G. D. Greenspan J. D. Dubner R. Maixner W. Bair E. (2018). Long-term changes in biopsychosocial characteristics related to temporomandibular disorder: findings from the OPPERA study. Pain 159, 2403–2413. 10.1097/j.pain.0000000000001348 30028791 PMC6193833

[B17] Groth C. Mol Debes N. Rask C. U. Lange T. Skov L. (2017). Course of tourette syndrome and comorbidities in a large prospective clinical study. J. Am. Acad. Child Adolesc. Psychiatry 56, 304–312. 10.1016/j.jaac.2017.01.010 28335874

[B18] Grove J. Ripke S. Als T. D. Mattheisen M. Walters R. K. Won H. (2019). Identification of common genetic risk variants for autism spectrum disorder. Nat. Genet. 51, 431–444. 10.1038/s41588-019-0344-8 30804558 PMC6454898

[B19] Han V. X. Patel S. Jones H. F. Dale R. C. (2021). Maternal immune activation and neuroinflammation in human neurodevelopmental disorders. Nat. Rev. Neurol. 17, 564–579. 10.1038/s41582-021-00530-8 34341569

[B20] He R. Mo J. Zhu K. Luo Q. Liu X. Huang H. (2023). The early life course-related traits with three psychiatric disorders: a two-sample Mendelian randomization study. Front. Psychiatry 14, 1098664. 10.3389/fpsyt.2023.1098664 37025349 PMC10070876

[B21] Jo K. B. Lee Y. J. Lee I. G. Lee S. C. Park J. Y. Ahn R. S. (2016). Association of pain intensity, pain-related disability, and depression with hypothalamus–pituitary–adrenal axis function in female patients with chronic temporomandibular disorders. Psychoneuroendocrinology 69, 106–115. 10.1016/j.psyneuen.2016.03.017 27082645

[B22] Jue H. Fang-fang L. Dan-fei C. Nuo C. Chun-lu Y. Ke-pin Y. (2023). A bidirectional Mendelian randomization study about the role of morning plasma cortisol in attention deficit hyperactivity disorder. Front. Psychiatry 14, 1148759. 10.3389/fpsyt.2023.1148759 37389173 PMC10303788

[B23] Kim C. Lee D. Y. Park J. Yang S.-J. Tan E. H. Alhambra D.-P. (2023). Safety outcomes of selective serotonin reuptake inhibitors in adolescent attention-deficit/hyperactivity disorder with comorbid depression: the ASSURE study – CORRIGENDUM. Psychol. Med. 53, 4831. 10.1017/s0033291723001022 37078398

[B24] Kurki M. I. Karjalainen J. Palta P. Sipilä T. P. Kristiansson K. Donner K. M. (2023). FinnGen provides genetic insights from a well-phenotyped isolated population. Nature 613, 508–518. 10.1038/s41586-022-05473-8 36653562 PMC9849126

[B25] Llorens M. Barba M. Torralbas J. Nadal R. Armario A. Gagliano H. (2022). Stress-related biomarkers and cognitive functioning in adolescents with ADHD: effect of childhood maltreatment. J. Psychiatric Res. 149, 217–225. 10.1016/j.jpsychires.2022.02.041 35287052

[B26] Lövgren A. Häggman‐Henrikson B. Visscher C. M. Lobbezoo F. Marklund S. Wänman A. (2015). Temporomandibular pain and jaw dysfunction at different ages covering the lifespan – a population based study. Eur. J. Pain 20, 532–540. 10.1002/ejp.755 26311138

[B27] Momany A. M. Kamradt J. M. Nikolas M. A. (2018). A meta-analysis of the association between birth weight and attention deficit hyperactivity disorder. J. Abnorm. child Psychol. 46, 1409–1426. 10.1007/s10802-017-0371-9 29159441 PMC5962386

[B28] Mota-Veloso I. Celeste R. K. Fonseca C. P. Soares M. E. C. Marques L. S. Ramos-Jorge M. L. (2017). Effects of attention deficit hyperactivity disorder signs and socio-economic status on sleep bruxism and tooth wear among schoolchildren: structural equation modelling approach. Int. J. Paediatr. Dent. 27, 523–531. 10.1111/ipd.12291 28155241

[B29] Ohrbach R. Bair E. Fillingim R. B. Gonzalez Y. Gordon S. M. Lim P. F. (2013). Clinical orofacial characteristics associated with risk of first-onset TMD: the OPPERA prospective cohort study. J. pain 14, T33–T50. 10.1016/j.jpain.2013.07.018 24275222 PMC3855658

[B30] Ribas M. O. Micai M. Caruso A. Fulceri F. Fazio M. Scattoni M. L. (2023). Technologies to support the diagnosis and/or treatment of neurodevelopmental disorders: a systematic review. Neurosci. Biobehav. Rev. 145, 105021. 10.1016/j.neubiorev.2022.105021 36581169

[B31] Robertson M. M. Eapen V. Singer H. S. Martino D. Scharf J. M. Paschou P. (2017). Gilles de la Tourette syndrome. Nat. Rev. Dis. Prim. 3, 16097. 10.1038/nrdp.2016.97 28150698

[B32] Salinas Fredricson A. Krüger Weiner C. Adami J. Rosén A. Lund B. Hedenberg-Magnusson B. (2022). The role of mental health and behavioral disorders in the development of temporomandibular disorder: a SWEREG-TMD nationwide case-control study. J. pain Res. 15, 2641–2655. 10.2147/JPR.S381333 36097536 PMC9464023

[B33] Schiffman E. Ohrbach R. Truelove E. Look J. Anderson G. Goulet J. P. (2014). Diagnostic criteria for temporomandibular disorders (DC/TMD) for clinical and research applications: recommendations of the international RDC/TMD Consortium network* and orofacial pain special interest group. J. Oral Facial Pain Headache 28, 6–27. 10.11607/jop.1151 24482784 PMC4478082

[B34] Shrivastava M. Battaglino R. Ye L. (2021). A comprehensive review on biomarkers associated with painful temporomandibular disorders. Int. J. Oral Sci. 13, 23. 10.1038/s41368-021-00129-1 34326304 PMC8322104

[B35] Slade G. D. Ohrbach R. Greenspan J. D. Fillingim R. B. Bair E. Sanders A. E. (2016). Painful temporomandibular disorder: decade of discovery from OPPERA studies. J. Dent. Res. 95, 1084–1092. 10.1177/0022034516653743 27339423 PMC5004239

[B36] Slade G. D. Sanders A. E. Bair E. Brownstein N. Dampier D. Knott C. (2013). Preclinical episodes of orofacial pain symptoms and their association with health care behaviors in the OPPERA prospective cohort study. Pain 154, 750–760. 10.1016/j.pain.2013.01.014 23531476 PMC3652580

[B37] Stelcer B. Sójka-Makowska A. Trzeszczyńska N. Samborska J. Teusz G. Pryliński M. (2022). Relationship between attention deficit hyperactivity disorder and temporomandibular disorders in adults: a questionnaire-based report. Eur. Rev. Med. Pharmacol. Sci. 26, 3858–3871. 10.26355/eurrev_202206_28953 35731055

[B38] Svensson P. Kumar A. (2016). Assessment of risk factors for oro‐facial pain and recent developments in classification: implications for management. J. Oral Rehabilitation 43, 977–989. 10.1111/joor.12447 27690281

[B39] Thapar A. Cooper M. (2016). Attention deficit hyperactivity disorder. Lancet London, Engl. 387, 1240–1250. 10.1016/S0140-6736(15)00238-X 26386541

[B40] van der Valk R. J. Kreiner-Møller E. Kooijman M. N. Guxens M. Stergiakouli E. Sääf A. (2015). A novel common variant in DCST2 is associated with length in early life and height in adulthood. Hum. Mol. Genet. 24, 1155–1168. 10.1093/hmg/ddu510 25281659 PMC4447786

[B41] Wang X. Yang Y. Lin L. Yao Q. Zhang J. (2023). Obesity and temporomandibular joint disorders: a systematic review and meta-analysis. BMC oral health 23, 607. 10.1186/s12903-023-03322-2 37644424 PMC10466750

[B42] Warrington N. M. Beaumont R. N. Horikoshi M. Day F. R. Helgeland Ø. Laurin C. (2019). Maternal and fetal genetic effects on birth weight and their relevance to cardio-metabolic risk factors. Nat. Genet. 51, 804–814. 10.1038/s41588-019-0403-1 31043758 PMC6522365

[B43] Wieckiewicz M. Boening K. Wiland P. Shiau Y.-Y. Paradowska-Stolarz A. (2015). Reported concepts for the treatment modalities and pain management of temporomandibular disorders. J. Headache Pain 16, 106. 10.1186/s10194-015-0586-5 26644030 PMC4671990

[B44] Winocur-Arias O. Amitai B.-C. Winocur E. Shmuly T. Grinstein Koren O. Reiter S. (2023). The prevalence of bruxism and oral parafunction activities among Israeli juveniles with autism spectrum disorder: a preliminary study during the COVID-19 pandemic. Cranio®, 1–9. 10.1080/08869634.2023.2277618 37964571

[B45] Yao C. Zhang Y. Lu P. Xiao B. Sun P. Tao J. (2023). Exploring the bidirectional relationship between pain and mental disorders: a comprehensive Mendelian randomization study. J. Headache Pain 24, 82. 10.1186/s10194-023-01612-2 37415130 PMC10326936

[B46] Yu D. Sul J. H. Tsetsos F. Nawaz M. S. Huang A. Y. Zelaya I. (2019). Interrogating the genetic determinants of tourette’s syndrome and other tic disorders through genome-wide association studies. Am. J. Psychiatry 176, 217–227. 10.1176/appi.ajp.2018.18070857 30818990 PMC6677250

[B47] Zablotsky B. Black L. I. Maenner M. J. Schieve L. A. Danielson M. L. Bitsko R. H. (2019). Prevalence and trends of developmental disabilities among children in the United States: 2009-2017. Pediatrics 144, e20190811. 10.1542/peds.2019-0811 31558576 PMC7076808

