# Supplementary material for: Neurodevelopmental disorders as a risk factor for temporomandibular disorder: evidence from Mendelian randomization studies
Source: Front Genet. 2024 Mar 8;15:1365596. doi: 10.3389/fgene.2024.1365596 (PMC10957778; doi:10.3389/fgene.2024.1365596)
Supplement: Supplementary file 1 [file DataSheet1.PDF]

Catalogue for supplementary figure:

The leave one out plot of the impact of Neurodevelopmental disorders on TMD…………….………….1
The funnel plot of the impact of Neurodevelopmental disorders on TMD…………….…………………2
The scatter plot of the impact of Neurodevelopmental disorders on TMD……….……………………3
The leave one out plot of the impact of TMD on Neurodevelopmental disorders ……………….………4
The funnel plot of the impact of TMD on Neurodevelopmental disorders………………………………5
The scatter plot of the impact of TMD on Neurodevelopmental disorders………………………………6

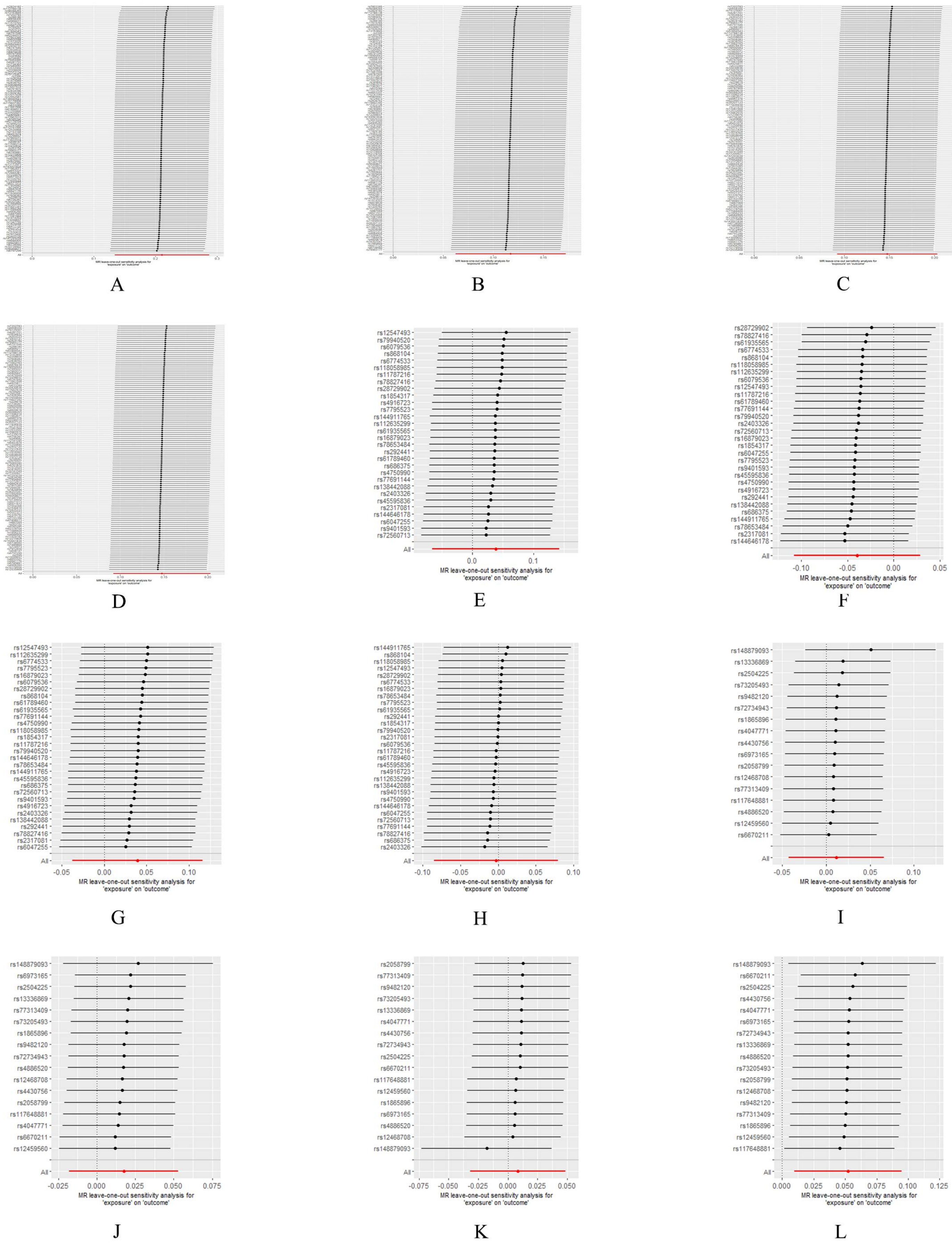

THE LEAVE ONE OUT PLOT OF THE EFFECT OF PSYCHIATRY DISORDER TO TMD
E.G(EXPOSURE-OUTCOME)
A: (ADHD-TEMPOROMANDIBULAR JOINT DISORDERS);
B: (ADHD-TEMPOROMANDIBULAR JOINT DISORDERS, INCLUDING AVOHILMO);
C: (ADHD-TMD RELATED PAIN);
D: (ADHD-TMD MUSCULAR PAIN LINKED WITH FIBROMYALGIA);
E : (ASD-TEMPOROMANDIBULAR JOINT DISORDERS);
F: (ASD-TEMPOROMANDIBULAR JOINT DISORDERS, INCLUDING AVOHILMO);
G: (ASD-TMD RELATED PAIN);
H: (TS-TMD MUSCULAR PAIN LINKED WITH FIBROMYALGIA);
I: (TS-TEMPOROMANDIBULAR JOINT DISORDERS);
J: (TS-TEMPOROMANDIBULAR JOINT DISORDERS, INCLUDING AVOHILMO);
K: (TS-TMD RELATED PAIN);
L: (TS-TMD MUSCULAR PAIN LINKED WITH FIBROMYALGIA)

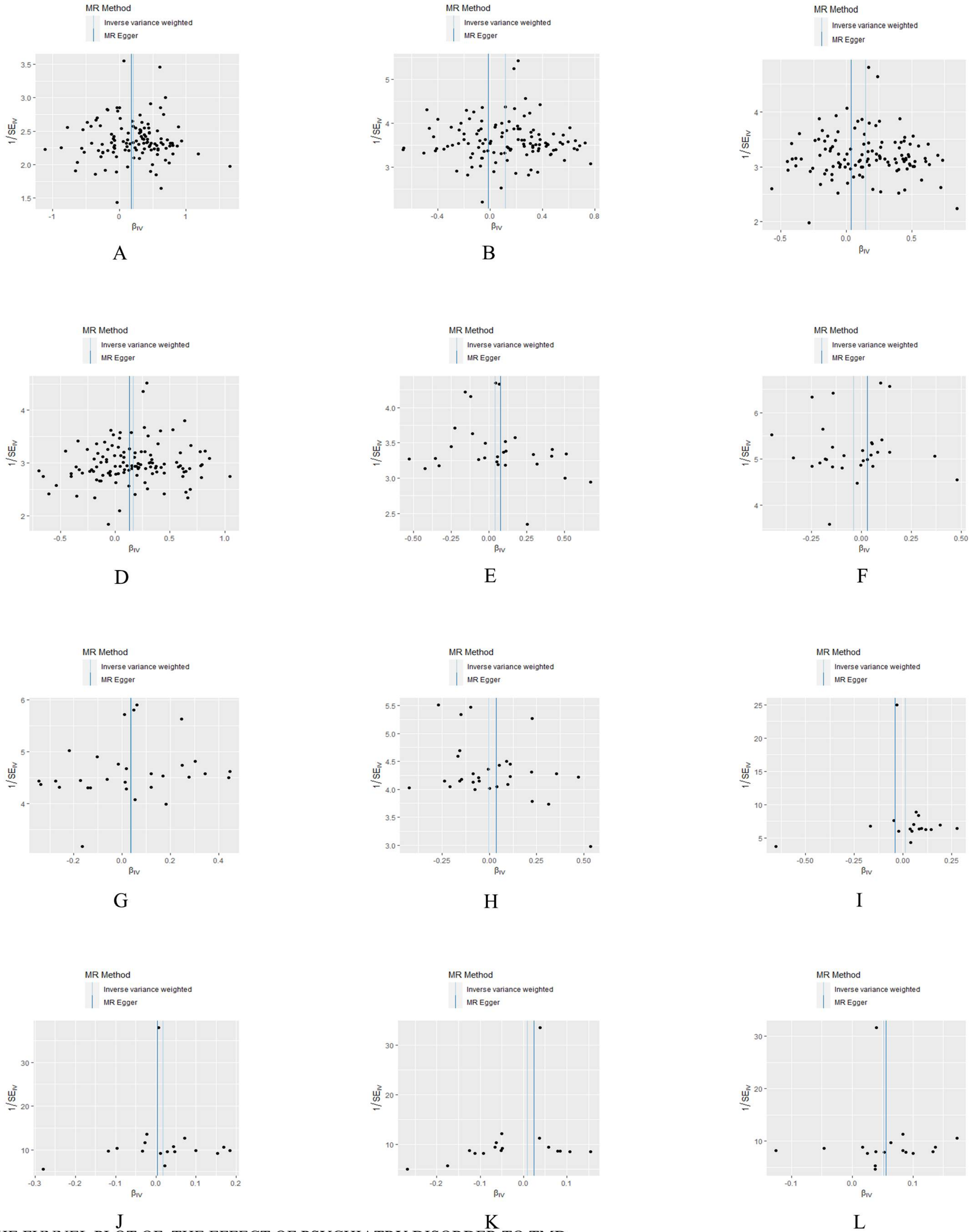

THE FUNNEL PLOT OF THE EFFECT OF PSYCHIATRY DISORDER TO TMD

E.G(EXPOSURE-OUTCOME)

A: (ADHD-TEMPOROMANDIBULAR JOINT DISORDERS);

B: (ADHD-TEMPOROMANDIBULAR JOINT DISORDERS, INCLUDING AVOHILMO);

C: (ADHD-TMD RELATED PAIN);

D: (ADHD-TMD MUSCULAR PAIN LINKED WITH FIBROMYALGIA);

E : (ASD-TEMPOROMANDIBULAR JOINT DISORDERS);

F: (ASD-TEMPOROMANDIBULAR JOINT DISORDERS, INCLUDING AVOHILMO);

G: (ASD-TMD RELATED PAIN);

H: (TS-TMD MUSCULAR PAIN LINKED WITH FIBROMYALGIA);

I: (TS-TEMPOROMANDIBULAR JOINT DISORDERS);

J: (TS-TEMPOROMANDIBULAR JOINT DISORDERS, INCLUDING AVOHILMO);

K: (TS-TMD RELATED PAIN);

L: (TS-TMD MUSCULAR PAIN LINKED WITH FIBROMYALGIA)

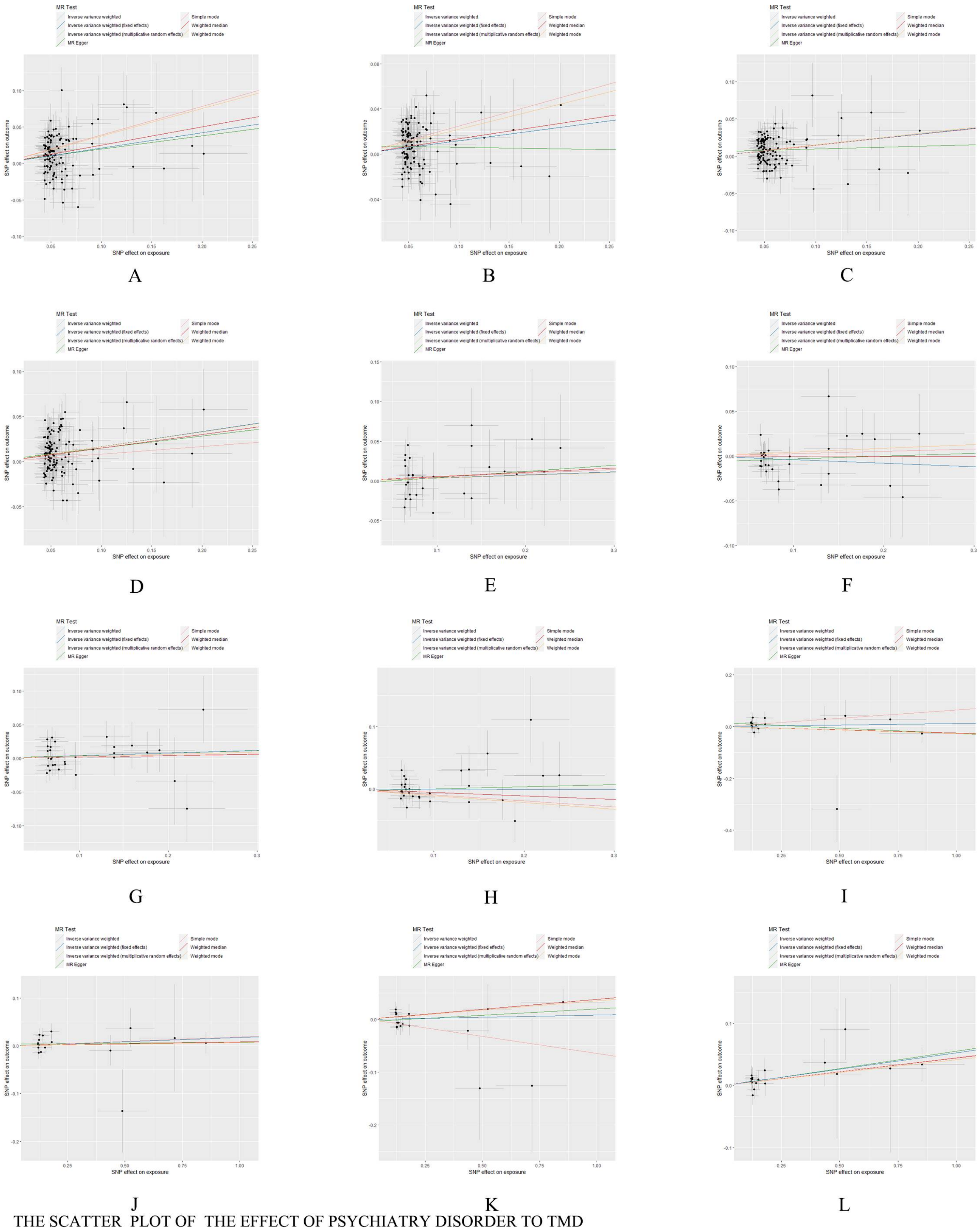

THE SCATTER PLOT OF THE EFFECT OF PSYCHIATRY DISORDER TO TMD
E.G(EXPOSURE-OUTCOME)
A: (ADHD-TEMPOROMANDIBULAR JOINT DISORDERS);
B: (ADHD-TEMPOROMANDIBULAR JOINT DISORDERS, INCLUDING AVOHILMO);
C: (ADHD-TMD RELATED PAIN);
D: (ADHD-TMD MUSCULAR PAIN LINKED WITH FIBROMYALGIA);
E : (ASD-TEMPOROMANDIBULAR JOINT DISORDERS);
F: (ASD-TEMPOROMANDIBULAR JOINT DISORDERS, INCLUDING AVOHILMO);
G: (ASD-TMD RELATED PAIN);
H: (TS-TMD MUSCULAR PAIN LINKED WITH FIBROMYALGIA);
I: (TS-TEMPOROMANDIBULAR JOINT DISORDERS);
J: (TS-TEMPOROMANDIBULAR JOINT DISORDERS, INCLUDING AVOHILMO);
K: (TS-TMD RELATED PAIN);
L: (TS-TMD MUSCULAR PAIN LINKED WITH FIBROMYALGIA)

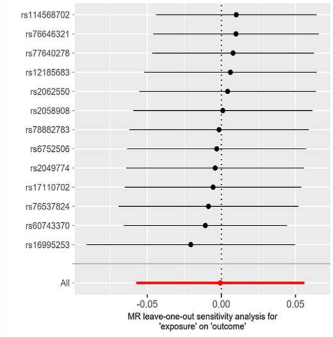

A

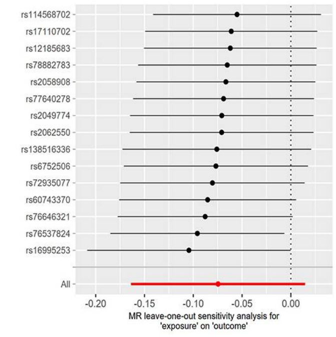

B

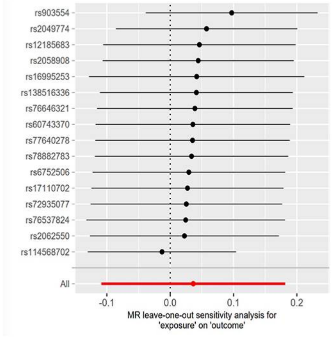

C

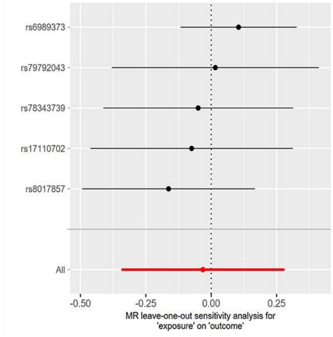

D

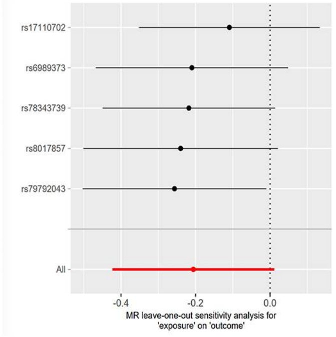

E

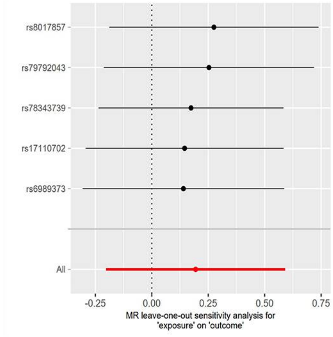

F

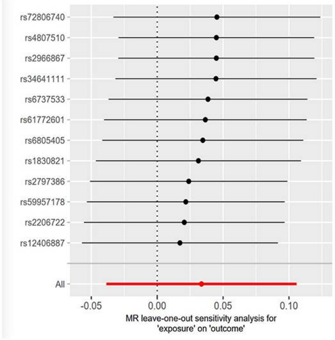

G

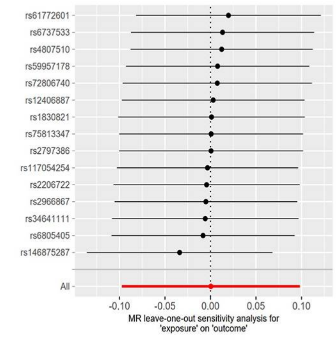

H

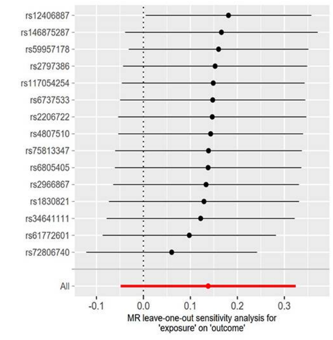

I

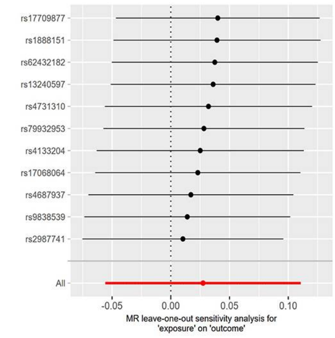

J

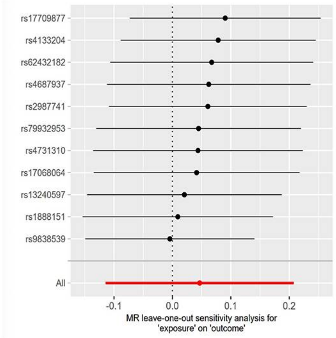

K

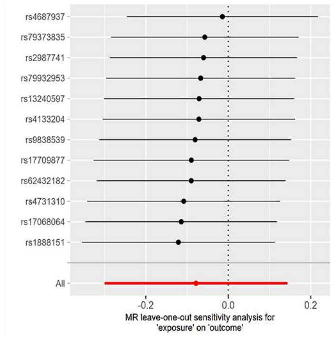

L

THE LEAVE ONE OUT PLOT OF THE EFFECT OF TMD TO PSYCHIATRY DISORDER
E.G(EXPOSURE-OUTCOME)
A: (TEMPOROMANDIBULAR JOINT DISORDERS-ADHD);
B: (TEMPOROMANDIBULAR JOINT DISORDERS-ASD);
C: (TEMPOROMANDIBULAR JOINT DISORDERS-TS);
D: (TEMPOROMANDIBULAR JOINT DISORDERS, INCLUDING AVOHILMO-ADHD);
E : (TEMPOROMANDIBULAR JOINT DISORDERS, INCLUDING AVOHILMO-ASD);
F: (TEMPOROMANDIBULAR JOINT DISORDERS, INCLUDING AVOHILMO-TS);
G: (TMD RELATED PAIN-ADHD);
H: (TMD RELATED PAIN-ASD);
I: (TMD RELATED PAIN-TS);
J: (TMD MUSCULAR PAIN LINKED WITH FIBROMYALGIA-ADHD);
K: (TMD MUSCULAR PAIN LINKED WITH FIBROMYALGIA-ASD);
L: (TMD MUSCULAR PAIN LINKED WITH FIBROMYALGIA-TS)

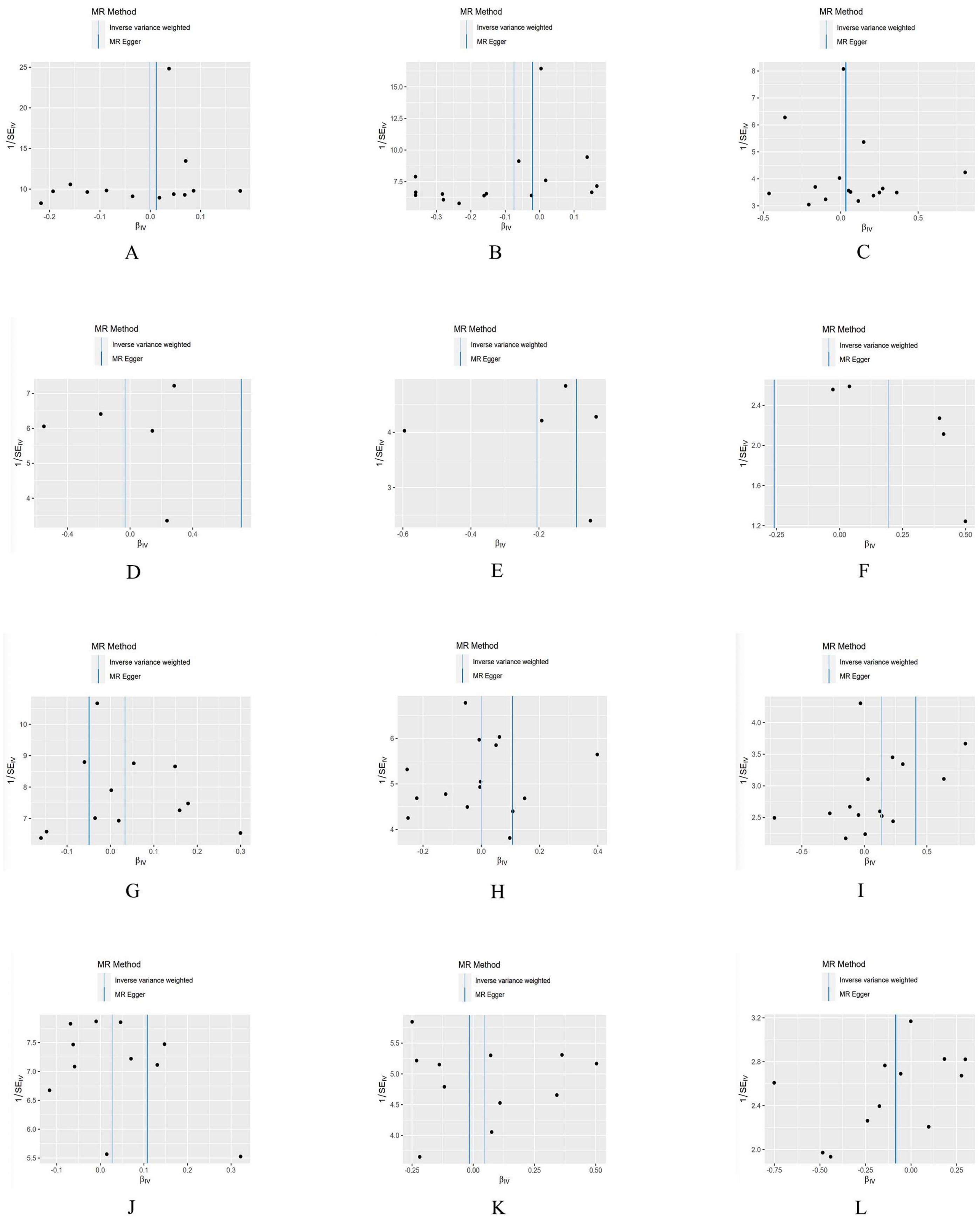

THE FUNNEL PLOT OF THE EFFECT OF TMD TO PSYCHIATRY DISORDER

E.G(EXPOSURE-OUTCOME)

A: (TEMPOROMANDIBULAR JOINT DISORDERS-ADHD);

B: (TEMPOROMANDIBULAR JOINT DISORDERS-ASD);

C: (TEMPOROMANDIBULAR JOINT DISORDERS-TS);

D: (TEMPOROMANDIBULAR JOINT DISORDERS, INCLUDING AVOHILMO-ADHD);

E : (TEMPOROMANDIBULAR JOINT DISORDERS, INCLUDING AVOHILMO-ASD);

F: (TEMPOROMANDIBULAR JOINT DISORDERS, INCLUDING AVOHILMO-TS);

G: (TMD RELATED PAIN-ADHD);

H: (TMD RELATED PAIN-ASD);

I: (TMD RELATED PAIN-TS);

J: (TMD MUSCULAR PAIN LINKED WITH FIBROMYALGIA-ADHD);

K: (TMD MUSCULAR PAIN LINKED WITH FIBROMYALGIA-ASD);

L: (TMD MUSCULAR PAIN LINKED WITH FIBROMYALGIA-TS)

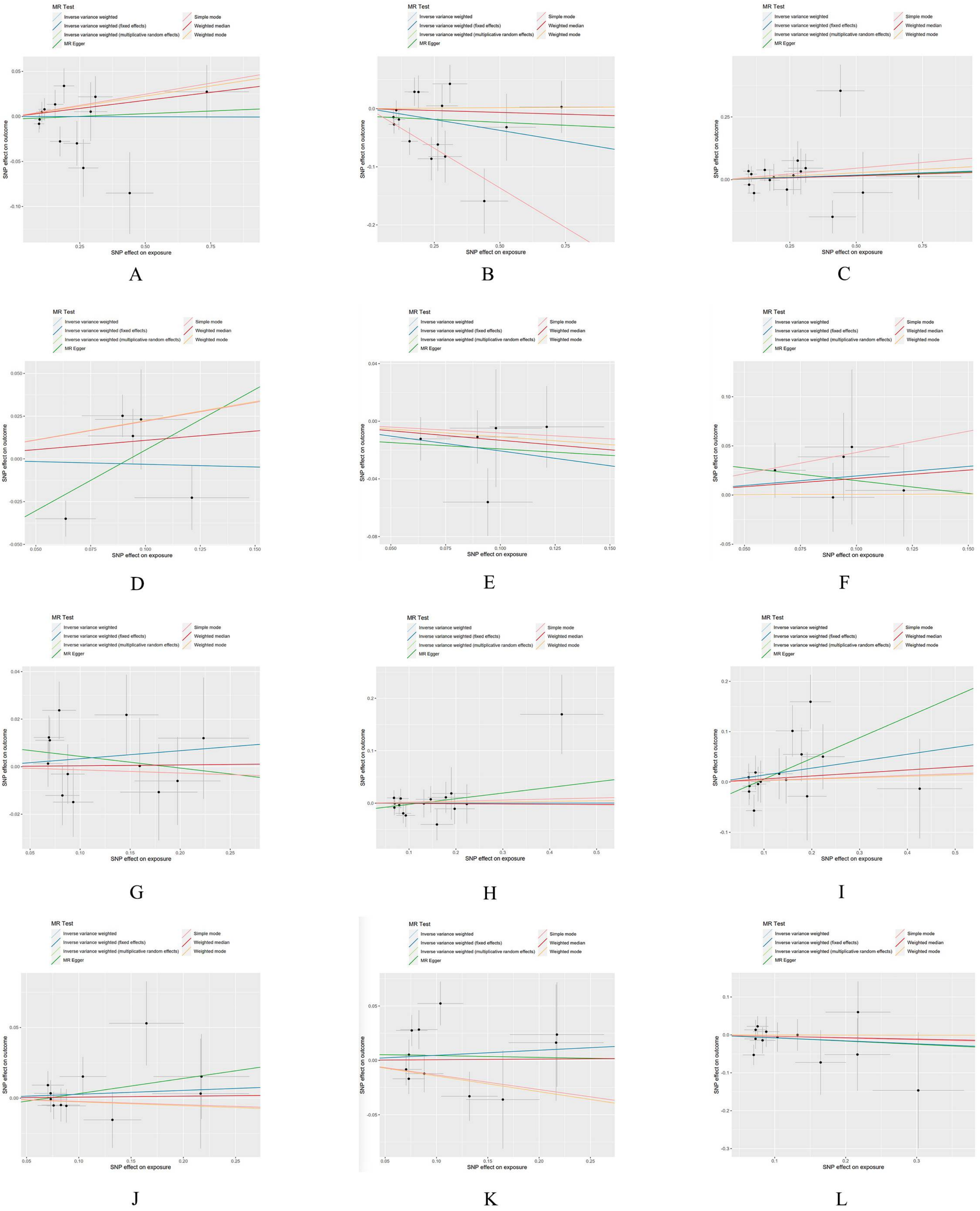

THE SCATTER PLOT OF THE EFFECT OF TMD TO PSYCHIATRY DISORDER
E.G(EXPOSURE-OUTCOME)
A: (TEMPOROMANDIBULAR JOINT DISORDERS-ADHD);
B: (TEMPOROMANDIBULAR JOINT DISORDERS-ASD);
C: (TEMPOROMANDIBULAR JOINT DISORDERS-TS);
D: (TEMPOROMANDIBULAR JOINT DISORDERS, INCLUDING AVOHILMO-ADHD);
E : (TEMPOROMANDIBULAR JOINT DISORDERS, INCLUDING AVOHILMO-ASD);
F: (TEMPOROMANDIBULAR JOINT DISORDERS, INCLUDING AVOHILMO-TS);
G: (TMD RELATED PAIN-ADHD);
H: (TMD RELATED PAIN-ASD);
I: (TMD RELATED PAIN-TS);
J: (TMD MUSCULAR PAIN LINKED WITH FIBROMYALGIA-ADHD);
K: (TMD MUSCULAR PAIN LINKED WITH FIBROMYALGIA-ASD);
L: (TMD MUSCULAR PAIN LINKED WITH FIBROMYALGIA-TS)
